# An unusually long appendix in a child: a case report

**DOI:** 10.4076/1757-1626-2-7398

**Published:** 2009-06-11

**Authors:** Ahmed Alzaraa, Sunil Chaudhry

**Affiliations:** Department of General Surgery, Leighton Hospital, Mid Cheshire Hospitals NHS Foundation TrustCrewe, CW1 4QJUK

## Abstract

**Introduction:**

Appendicitis is the most common surgical emergency, but its diagnosis in children can be a challenge to the treating surgeon.

**Case presentation:**

A 10 years-old girl was admitted to the paediatrics surgical ward with a right sided abdominal pain. The initial clinical presentation was vague. She underwent an appendicectomy which revealed a very long appendix.

**Conclusion:**

About half of the adult patients with appendicitis present with the known classical signs and symptoms, but the clinical scenario might be completely different in children. This case is educational as it highlights the importance of the atypical presentations of an unusually long appendix in children.

## Introduction

The well known clinical picture of appendicitis includes a right lower abdominal pain, nausea, vomiting and a high temperature. However, children may present with unusual signs and symptoms, making the diagnosis very difficult.

## Case presentation

**Figure 1.**
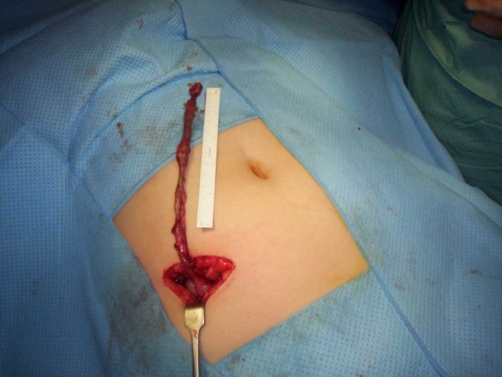
A long appendix before dissection measuring 17 cm.

**Figure 2.**
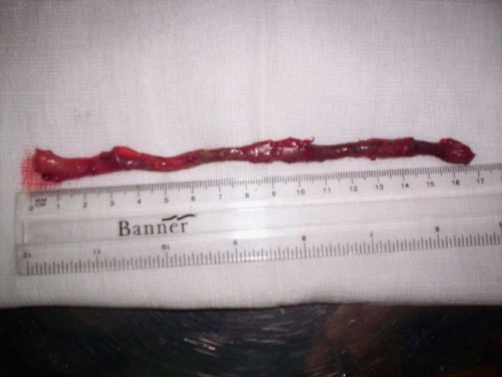
The long appendix after dissection.

A 10 years-old girl was admitted to the paediatric surgical ward for an acute abdominal pain which had been present for two days. The pain was constant, dull, on the right side of the abdomen, from the right upper quadrant to the right iliac fossa. She had vomited three times and her appetite was poor. Her bowel function was normal. On clinical examination, her pulse rate was 100/minute, blood pressure 128/72 mmHg and was febrile with a temperature of 37.5°C. She had a tender right upper quadrant and right iliac fossa. There was no guarding, rebound tenderness or rigidity. Blood tests showed total white cell count of 14.00 × 10*9/L, neutrophils 10.58 × 10*9/L, and C-reactive protein 53 mg/L. All other blood tests were normal. Urinalysis did not reveal anything significant. The clinical picture on initial examination was not very clear, so the child was observed and prescribed analgesia and intravenous fluids. Later in the night, she was still in pain. A decision was made to proceed for an appendicectomy. A muscle splitting Lanz incision was made in the right iliac fossa. The caecum was in the right iliac fossa. The appendix was swollen, inflamed and paracaecal in position. It was very long and after one coil on itself was going superiorly. At this stage the incision was converted to muscle cutting and was extended. The tip of the appendix reached the subhepatic area. After dividing the mesoappendix, it measured 17 cm in length (Figure 1 & 2). The appendicectomy was performed. Postoperatively the child did well. The histopathology report was of a fibrin covered gangrenous appendix with no perforation. The child went home after two days.

## Discussion

The appendix is a long, thin diverticulum arising from the inferior tip of the caecum. It is lined with colonic epithelium with interspersed submucosal lymphoid follicles. Its function is unknown, although its lymphatic tissue and secretion of immunoglobulins suggest that it may play a specialized role in the immune system [1]. The length and position of the appendix can vary considerably which many a times poses a diagnostic dilemma, especially in a young paediatric patient. Though the position of the base of the appendix in relation to the caecum is essentially constant (McBurney's point), the location of its free tip is highly variable [2]. It may be retrocaecal (28%-68%), pelvic (27%-53%) [3,4], paracaecal and paracolic, anterior or pre-ileal (1%), post-ileal, within a hernial sac (2%), or the caecum itself may lie in the subhepatic position because of the arrest of its descent (4%) [1]. Very rarely, the appendix may occupy a position in left upper or lower quadrant of the abdomen (0.1% each) [3,4] as a result of malrotation of the mid-gut. The average length of the appendix is 4.5 cm in neonates and 9.5 cm in adults [1], but this may vary between 2 cm to 20 cm [3]. The longest appendix reported in the literature measured 26 cm removed from 72 year old during an autopsy in Croatia in 2006 (Guinness World Records). Raschka S, et al. [5] found that the appendix length correlated highly significantly with body weight in his study of 167 patients who underwent appendicectomies. Fifty percent of adults present with the classical scenario of periumbilical pain, nausea, migration of pain to the right lower quadrant, and later vomiting with fever. This is less common in children as many of the presenting features in appendicitis are age-dependent [1]. Patients may present with severe diarrhoea, acute urinary retention, acute hemiscrotal pain, and priapism [2,6,7,8]. Paediatric patients are difficult to examine because they are often fearful of the examiner, cry or become uncooperative with the examination, or cannot convey what aggravates their pain or how the symptoms had progressed [9]. Arrested caecal descent occurs where the caecum lies in the subhepatic position but does not descend to the right iliac fossa. As a result of that, an inflammation of a subhepatic appendix can mimic cholecystitis, and perforation of a subhepatic appendix can mimic liver abscess [10]. Our young patient had an unusually long appendix of 17 cm. for her age. Although the caecum was in the right iliac fossa, the very long appendix after one coil on itself lied along the entire length of ascending colon with its tip reaching the subhepatic area, and just touching the under surface of the liver. This resulted in pain and tenderness along the whole of the right flank, from the right upper quadrant to the right iliac fossa. This did cause diagnostic uncertainty. With ultrasound scan not available out of hours, a CT scan of abdomen was not done to avoid radiation to the young child. However, raised inflammatory markers and keeping a low threshold for appendicectomies in children, prompted us to undertake an emergency appendicectomy. Had this long appendix lied coiled in the right iliac fossa, it would have presented with classical signs and symptoms and McBurney's point tenderness, or its tip could have lied far away in the left iliac fossa, central abdomen or even the left upper quadrant causing more diagnostic problems. The delay in making a diagnosis in a child may result in a perforation of the appendix with its dire consequences.

## Conclusion

Children may not present with the classical symptoms and signs of a right lower quadrant abdominal pain, nausea, vomiting, and a high temperature. A long appendix is associated with further diagnostic problems by virtue of its inflamed tip reaching a faraway location (e.g. right upper, left lower, rarely left upper quadrant of abdomen). The treating surgeon should bear this in mind, since a delay in diagnosing and making the decision to operate results in an increased risk of perforation and morbidity.

## List of abbreviations

CT: Computer Tomography
